# Nucleated red blood cell count as a novel predictive biomarker for acute kidney injury and prognosis in patients with acute pancreatitis: A retrospective cohort study

**DOI:** 10.1371/journal.pone.0330611

**Published:** 2025-08-22

**Authors:** Qiang Yong Yan, Xi Ming Mo, Wen Wu Cai, Yan Peng, Hua Lan Huang

**Affiliations:** 1 Department of Pharmacy, The Second Xiangya Hospital of Central South University, Changsha, Hunan, China; 2 Department of Laboratory Medicine, The Second Xiangya Hospital of Central South University, Changsha, Hunan, China; 3 Department of General Surgery, The Second Xiangya Hospital of Central South University, Changsha, Hunan, China; University of Nebraska Medical Center College of Medicine, UNITED STATES OF AMERICA

## Abstract

**Objectives:**

Nucleated red blood cells (NRBCs) have been reported to correlate to poor prognosis in critically ill patients. This study aimed to identify the role of NRBC count in early diagnosis of acute kidney injury (AKI) and prediction of poor prognosis in patients with acute pancreatitis (AP).

**Methods:**

This retrospective cohort study enrolled AP patients who were admitted to our hospital from January 1, 2020 to January 1, 2024. Demographic data, NRBC count, laboratory indicators, and outcomes were recorded. Binary logistic regression analysis was used to identify independent biomarkers for AKI diagnosis and prognosis of AP. Receiver operating characteristic (ROC) curves, net reclassification index (NRI) and integrated discrimination improvement (IDI) were used to evaluate the predictive value of NRBC count. Kaplan-Meier curves were generated to compare survival rate between different groups.

**Results:**

A total of 486 patients with AP were included in this study. Patients with NRBCs in their peripheral blood were classified into the NRBC (+) group (n = 190), and those without NRBCs in their peripheral blood were classified into the NRBC (-) group (n = 296). Patients in the NRBC (+) group had a higher AKI incidence (29.5% vs 3.0%, p < 0.001) and poor prognosis rate (12.7% vs 1.0%, p < 0.001) than patients in the NRBC (-) group. Binary logistic regression analysis showed that NRBC count was significantly associated with AKI incidence (OR = 3601.361, p < 0.05) and poor prognosis (OR = 204.434, p < 0.05) in AP patients. Area under the ROC curve (AUC) was 0.830 for NRBC count predicting AKI (cutoff value: 0/L) and 0.867 for NRBC count predicting poor prognosis in AP patients (cutoff value: 0.015 × 10^9^/L). Kaplan–Meier survival analysis demonstrated that patients with NRBC count > 0.015 × 10^9^/L (χ² = 85.09, p < 0.001) exhibited significantly lower survival rates during the 30-day follow-up period. NRBC count offered predictive performance comparable to procalcitonin (PCT) and outperformed C-reactive protein (CRP).

**Conclusion:**

NRBC count serves as a valuable predictive biomarker for both AKI incidence and poor prognosis in patients with AP.

## Background

In human, nucleated red blood cells (NRBCs) are mainly generated in the bone marrow. NRBCs are usually undetectable in normal peripheral blood in adults. However, NRBCs can be detected in the peripheral blood in the context of brisk hemolysis, rapid blood loss, blood cancer, extra-marrow hematopoiesis, inflammation, and severe hypoxia [1,2].

Acute pancreatitis (AP) as a risk factor for organ failure is a potentially life-threatening condition [3,4]. Patients with AP are susceptible to complications including acute kidney injury (AKI), which can significantly increase the mortality of patients. As reported, infection or sepsis may be the potential etiology for AKI in AP patients [5,6].

Recent studies have proposed that NRBC count can be used as a biomarker for sepsis and a valid mortality predictor in critically ill patients [7,8]. However, there are no studies that evaluate the role of NRBC count in AKI incidence or prognosis in patients with AP. This study aimed to determine the predictive role of NRBC count to guide early AKI diagnosis and poor prognosis in AP patients.

## Methods

### Study population

This retrospective cohort study was approved by the Ethics Committee of the Second Xiangya Hospital of Central South University (IRB No. LYEC2024-K0144). Researchers had no access to any information that would identify individual participants during or after data collection. The requirement for informed consent was waived. AP patients who were admitted to the Second Xiangya Hospital from January 1, 2020 to January 1, 2024 were included in this study. Clinical and laboratory data were extracted from the hospital’s electronic information capture system on August 24, 2024. AP was diagnosed and graded according to the revision of the Atlanta classification [9]. AP diagnosis requires meeting at least two of the following criteria: (1) abdominal pain consistent with AP; (2) level of serum amylase and/or lipase 3 times or more the upper limit of normal; (3) characteristic findings of AP on a computed tomography (CT) scan, magnetic resonance imaging, or transabdominal ultrasonography.

Exclusion criteria: (1) age < 18 years old; (2) pregnancy; (3) a history of blood disorders, including blood cancers; (4) chronic kidney disease, renal parenchymal or interstitial injury due to factors other than pancreatitis, or a history of renal transplantation; (5) incomplete or missing medical records; (6) death or discharge within 3 days of admission.

AKI was diagnosed during the period from admission to the end of treatment on the 7th day after admission, according to the Kidney Disease: Improving Global Outcomes (KDIGO) criteria [10]: serum creatinine (Crea) increases to 26.5 μmol/L within 48 h or by 50% within 7 days of admission (excluding urine output criteria).

### Clinical and laboratory data collection

Basic information included age, gender, smoking history, alcohol use, etiology, disease severity, underlying conditions, comorbidities, length of hospital stay, and outcomes. Patient outcomes included improvement, poor treatment response, and death. Poor treatment response and death were considered as poor prognosis. Mortality at 30 days was the primary endpoint. Patients who were discharged within this timeframe were also followed up by telephone to determine their outcome.

Laboratory data included coagulation indicators, such as prothrombin time (PT), activated partial thromboplastin time (APTT) and fibrinogen (Fib), tested by a Sysmex CS5100 coagulation analyzer (Japan); biochemical indicators, including serum Crea, total protein (TP) and albumin (ALB) that were measured by a Roche Cobas C702 (German), C-reactive protein (CRP) and procalcitonin (PCT) that were detected by Beckman IMMAGE 800 (USA) and Roche Cobas e801 (German), respectively; routine blood indicators, such as NRBC count, hemoglobin (Hb) and white blood cell count (WBC), measured using an automatic Sysmex XN hematology analyzer (Japan).

NRBCs were counted in a WBC & NRBC (WNR) channel using matched Lysercell WNR reagents and stains on the Sysmex XN hematology analyzer, through the technique of flow cytometry using a semiconductor laser. Rigorous internal quality control was performed prior to use. NRBC count was expressed as an absolute number of billion cells per liter (10^9^/L). The analytical performance of biomarkers should be validated before clinical use [11]. The performance of NRBC count analyzed with Sysmex XN was evaluated in many studies [12–14], and a better performance (correlation: r = 0.99 to 0.97) was found in comparison with that of microscopic differential counts performed according to the protocol of the National Committee for Clinical Laboratory Standards (NCCLS document H20-A) [12,13]. The intra-assay coefficient of variation (CV) ranged from 1.2% to 4.4% when 8 different levels of NRBCs were repeatedly analyzed (n = 20) with the Sysmex XN, demonstrating that the Sysmex XN analyzer showed high precision [13].

### Statistical analysis

Normally distributed data were expressed as means ± standard deviations (SD), and Student’s t-test was used for comparisons between groups. Data that did not follow a normal distribution were expressed as median and interquartile range (IQR), and non-parametric Mann-Whitney test was applied for comparisons between groups. Chi-square test was applied to compare categorical variables. All statistical tests were 2-sided, and p < 0.05 indicated statistical significance. Variance inflation factors (VIFs) were calculated to assess multicollinearity before regression analysis, and variables at the risk of multicollinearity were excluded. Univariate logistic regression followed by binary logistic regression was performed to identify factors associated with AKI incidence and patient prognosis. IBM SPSS Statistics 27 and R software (version 4.2.3) were used for statistical analyses. Receiver operating characteristic (ROC) curves, area under the ROC curve (AUC), net reclassification index (NRI), and integrated discrimination improvement (IDI) were used to evaluate the predictive value of NRBC count. Kaplan-Meier curves (log-rank test) were generated to compare survival rate between different groups. ROC and Kaplan-Meier curves were established by GraphPad Prism 8 (GraphPad Software Inc., USA).

## Results

### Demographic and laboratory characteristics of patients

**Fig 1:**
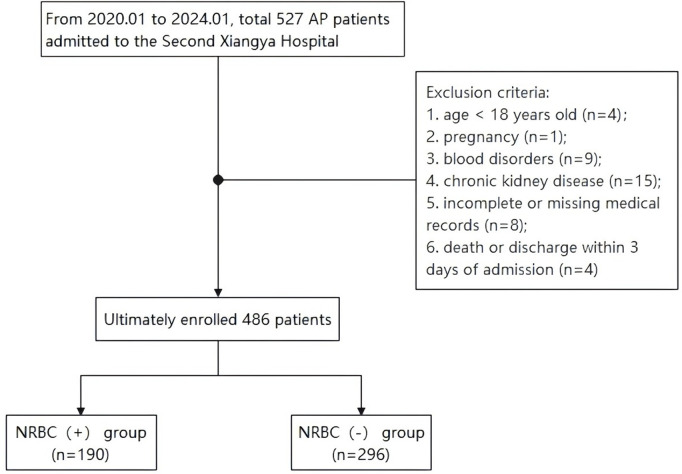
Flowchart of study participants. AP, acute pancreatitis; NRBC (+), presence of nucleated red blood cells in the peripheral blood on admission; NRBC (-), absence of nucleated red blood cells in the peripheral blood on admission.

**Fig 2:**
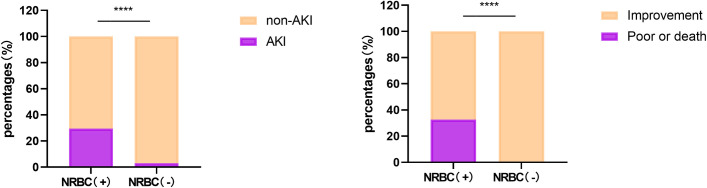
Comparison of AKI incidence and poor prognosis between NRBC (+) and NRBC (-) groups. AKI, acute kidney injury; NRBC (+), presence of nucleated red blood cells in the peripheral blood on admission; NRBC (-), absence of nucleated red blood cells in the peripheral blood on admission; **** = p < 0.001 according to the chi-square test.

This clinical trial finally enrolled 486 AP patients (Fig 1), including 303 males and 183 females, with a mean age of 49.0 years (range: 18–91 years). According to the presence of NRBCs in the peripheral blood, the participants were classified into two groups: NRBC (+) group (n = 190), and NRBC (-) group (n = 296). Demographic data of the patients are shown in Table 1. There were no significant differences between the NRBC (+) and NRBC (-) groups in terms of gender, age, smoking history, use of vasoactive agent, or surgery during hospitalization. Significant differences were found between the two groups in etiology of pancreatitis, chronic obstructive pulmonary disease (COPD), hypertension, alcohol use, hemodialysis during hospitalization, and severity of pancreatitis (p < 0.05). As compared to the NRBC (-) group, the NRBC (+) group demonstrated a higher incidence of multiple organ dysfunction syndrome (MODS; 0.3% vs 6.8%, p < 0.001), acute respiratory distress syndrome (ARDS; 0.3% vs 5.3%, p < 0.001), sepsis (1.0% vs 4.7%, p = 0.01), and shock (0.7% vs 7.4%, p < 0.001). Patients in the NRBC (+) group were more prone to develop AKI (29.5% vs 3.0%, p < 0.001) and have poor prognosis (12.7% vs 1.0%, p < 0.001) than patients in the NRBC (-) group (Fig 2).

**Table 1:** Demographic characteristics of study participants.

	NRBC (+) (n = 190)	NRBC (-) (n = 296)	p – value
**Age (years)**	49.5 ± 15.2	48.8 ± 14.9	0.626
**Gender**			
** Male, n(%)**	115 (60.5)	188 (63.5)	0.507
**Etiology of pancreatitis, n(%)**			0.034
** Gallbladder Diseases**	52 (27.4)	103 (34.8)	
** Hyperglycaemia**	51 (26.8)	47 (15.9)	
** Alcohol consumption**	4 (2.1)	7 (2.4)	
** Others**	83 (43.7)	139 (47.0)	
**Basic disease, n(%)**			
** COPD**	18 (9.5)	0 (0)	< 0.001
** Diabetes mellitus**	40 (21.1)	53 (17.9)	0.389
** Hypertension**	53 (27.9)	49 (16.6)	0.003
** Coronary heart disease**	10 (5.3)	14 (4.7)	0.791
** Fatty liver**	40 (21.1)	72 (24.3)	0.403
**Unhealthy living habits, n(%)**			
** History of smoking**	69 (37.1)	85 (28.8)	0.058
** History of alcohol intake**	69 (37.1)	78 (26.4)	0.013
**Use of vasoactive agent, n (%)**	11 (5.8)	13 (4.4)	0.493
**Use of hemopurification, n (%)**	11 (5.8)	1 (0.3)	< 0.001
**Surgery during hospitalization, n (%)**	22 (11.6)	20 (6.8)	0.067
**Severity of pancreatitis, n (%)**			< 0.001
** Mild**	75 (39.5)	230 (77.7)	
** Moderate**	43 (22.6)	56 (18.9)	
** Severe**	72 (37.9)	10 (3.4)	
**ICU treatment, n(%)**	61 (32.1)	15 (5.1)	< 0.001
**Length of hospital stay, days**	11 (7, 20)	7 (5, 10)	< 0.001
**AKI, n(%)**	56 (29.5)	9 (3.0)	< 0.001
**MODS, n(%)**	13 (6.8)	1 (0.3)	< 0.001
**ARDS, n(%)**	10 (5.3)	1 (0.3)	< 0.001
**Sepsis, n(%)**	9 (4.7)	3 (1.0)	0.010
**Shock, n(%)**	14 (7.4)	2 (0.7)	< 0.001
**Prognosis, n (%)**			< 0.001
** Poor or death**	24 (12.7)	3 (1.0)	
** Improvement**	165 (87.3)	292 (99.0)	

NRBC (+), presence of nucleated red blood cells in the peripheral blood on admission; NRBC (-), absence of nucleated red blood cells in the peripheral blood on admission; AP, acute pancreatitis; COPD, chronic obstructive pulmonary disease; ICU, intensive care unit; AKI, acute kidney injury; MODS, multiple organ dysfunction syndrome; ARDS, acute respiratory distress syndrome.

Admission laboratory indicators varied significantly between NRBC (+) and NRBC (-) groups (Table 2). In terms of coagulation indicators, PT, APTT, Fib, and D-dimer in the NRBC (+) group were significantly higher than in the NRBC (-) group, whereas antithrombin (AT) in the NRBC (+) group was remarkably lower (p < 0.001). Among routine blood indicators, WBC, neutrophil count, red blood cell distribution width (RDW) -coefficient of variation (RDW-CV), and RDW-standard deviation (RDW-SD) in the NRBC (+) group showed significantly higher levels than in the NRBC (-) group, while Hb and platelet count (PLT) in the NRBC (+) group were reversely lower (p < 0.05). With regard to biochemical indicators, aspartate aminotransferase (AST), total bilirubin (TBIL), direct bilirubin (DBIL), blood urea nitrogen (Urea), Crea, blood glucose (GLU), lactic acid (LAC), lactate dehydrogenase (LDH), triglyceride (TG), and low-density lipoprotein (LDL) in the NRBC (+) group were significantly higher than in the NRBC (-) group; in contrast, TP, ALB, and high-density lipoprotein (HDL) in the NRBC (+) group were lower (p < 0.001). Additionally, inflammatory indicators, PCT and CRP, were significantly higher in the NRBC (+) group than those in the NRBC (-) group (p < 0.001). No significant differences were noted between the two groups regarding thrombin time (TT), alanine aminotransferase (ALT), uric acid (UA), serum lipase (LPS), serum amylase (AMY), and total cholesterol (CHOL).

**Table 2:** Comparison of laboratory indicators on admission between NRBC (+) and NRBC (-) groups.

	NRBC (+) (n = 190)	NRBC (-) (n = 296)	p – value
**PT, s**	14.1 (11.8, 13.9)	12.7 (12.8, 15.1)	< 0.001
**APTT, s**	36.4 (30.3, 42.1)	32.5 (28.0, 38.3)	< 0.001
**Fibrinogen, g/L**	5.9 (4.1, 7.3)	4.4 (3.3, 5.8)	< 0.001
**TT, s**	16.9 (15.8, 18.1)	16.7 (15.7, 17.8)	0.092
**D-dimer, μg/mL FEU**	3.8 (2.0, 8.3)	1.4 (0.6, 3.3)	< 0.001
**Antithrombin, %**	80.1 (70, 93.3)	91 (79.3, 100.5)	< 0.001
**WBC, × 10** ^**9**^ **/L**	12.2 (8.4, 16.2)	9.4 (6.1, 13.0)	< 0.001
**Hemoglobin, g/L**	126 (104, 143)	130 (116, 145)	0.034
**Platelet count, × 10** ^**9**^ **/L**	203 (147, 271)	217 (171, 283)	0.041
**Neutrophil, × 10** ^**9**^ **/L**	10.5 (6.9, 14.8)	7.6 (4.3, 11.3)	< 0.001
**RDW-CV, %**	13.6 (13.0, 14.4)	13.0 (12.5, 13.7)	< 0.001
**RDW-SD, fl**	45.8 (42.9, 48.5)	43.4 (41.1, 45.9)	< 0.001
**ALT, U/L**	27.3 (14.0, 76.2)	26.4 (15.6, 69.2)	0.844
**AST, U/L**	38.4 (23.0, 70.7)	26.6 (17.0, 50.5)	< 0.001
**Total protein, g/L**	60.3 ± 9.2	64.5 ± 9.3	< 0.001
**Albumin, g/L**	34.3 (29.6, 38.7)	37.9 (34.6, 42.4)	< 0.001
**Total bilirubin, μmol/L**	17.1 (10.6, 31.1)	14.2 (9.6, 23.7)	0.034
**Direct bilirubin, μmol/L**	6.6 (4.0, 13.4)	5.4 (3.3, 10.0)	0.010
**Urea, mmol/L**	6.7 (4.1, 11.4)	4.5 (3.4, 6.0)	< 0.001
**Crea, μmol/L**	76.0 (59.6, 123.4)	68.9 (54.3, 80.1)	< 0.001
**Uric acid, μmol/L**	291.5 (197.8, 417.9)	286.9 (223.0, 373.0)	0.698
**GLU, mmol/L**	8.1 (5.9, 13.9)	5.7 (4.7, 8.5)	< 0.001
**Serum lipase, U/L**	192.9 (68.7, 739.7)	145.3 (63.6, 395.6)	0.092
**Serum amylase, U/L**	137.0 (41.9, 489.0)	99.9 (45.1, 298.6)	0.065
**LAC, mmol/L**	3.11 (2.46, 3.64)	1.77 (1.34, 2.25)	0.002
**LDH, U/L**	479.5 (297.9, 1055.6)	237.8 (190.4, 310.0)	< 0.001
**Triglyceride, mmol/L**	2.6 (1.3, 8.0)	1.6 (1.0, 3.7)	< 0.001
**Total cholesterol, mmol/L**	4.5 (3.3, 5.9)	4.5 (3.6, 5.6)	0.809
**HDL, mmol/L**	0.7 (0.4, 1.1)	0.9 (0.6, 1.2)	<0.001
**LDL, mmol/L**	2.5 (1.6, 3.3)	1.8 (1.0, 2.9)	<0.001
**CRP, mg/L**	154.0 (75.9, 306.5)	39.9 (9.1, 114.8)	<0.001
**PCT, ng/mL**	1.42 (0.34, 5.57)	0.19 (0.08, 0.43)	<0.001

NRBC (+), presence of nucleated red blood cells in the peripheral blood on admission; NRBC (-), absence of nucleated red blood cells in the peripheral blood on admission; PT, prothrombin time; APTT, active partial thromboplastin time; TT, thrombin time; WBC, white blood cell count; RDW-CV, red blood cell volume distribution width – coefficient of variation; RDW-SD, red blood cell volume distribution width-standard deviation; ALT, alanine aminotransferase; AST, aspartate aminotransferase; Urea, blood urea nitrogen; Crea, serum creatinine; GLU, blood glucose; LAC, lactic acid; LDH, lactate dehydrogenase; HDL, high-density lipoprotein; LDL, low-density lipoprotein; CRP, C-reactive protein; PCT, procalcitonin.

### Risk factors for AKI and poor prognosis in AP patients

Univariate logistic regression was performed to identify rick factors for AKI. NRBC count, PT, APTT, D-dimer, AT, PLT, RDW-CV, RDW-SD, AST, TP, ALB, TG, CHOL, PCT, and CRP were found to show a significant relationship with the incidence of AKI in AP patients. The variables with statistical significance were subject to binary logistic regression analysis. To ensure the accuracy and stability, VIFs were calculated to explore the potential multicollinearity. Typically, VIF > 10 indicates severe multicollinearity. As shown in Table 3, all variables had a VIF value below 3, suggesting a low risk of multicollinearity. The results of binary logistic regression analysis revealed that NRBC count (OR = 3601.361, p < 0.05) and PCT level (OR = 1.062, p < 0.05) were independent predictors of AKI in patients with AP (Table 3).

**Table 3:** Binary logistic regression analysis for risk factors associated with AKI in AP patients.

Variables	B	Wald	OR (95% CI)	p – value	VIF
**NRBC, × 10** ^**9**^ **/L**	8.189	5.312	3601.361 (3.405, 3809284.782)	0.021	2.320
**PT, s**	−0.039	0.086	0.962 (0.741, 1.249)	0.770	1.611
**APTT, s**	0.035	0.461	1.035 (0.936, 1.145)	0.497	2.400
**D-dimer, μg/mL FEU**	−0.037	0.187	0.963 (0.814, 1.141)	0.666	2.370
**Antithrombin, %**	0.013	0.186	1.013 (0.955, 1.075)	0.667	2.001
**Platelet count, × 10** ^**9**^ **/L**	−0.001	0.041	0.999 (0.988, 1.010)	0.839	1.319
**RDW-CV, %**	0.284	0.846	1.328 (0.726, 2.430)	0.358	1.749
**RDW-SD, fl**	0.107	1.507	1.113 (0.938, 1.321)	0.220	1.660
**AST, U/L**	0.001	0.150	1.001 (0.995, 1.008)	0.699	1.456
**Total protein, g/L**	−0.109	2.011	0.897 (0.771, 1.043)	0.156	2.173
**Albumin, g/L**	−0.030	0.391	0.971 (0.884, 1.066)	0.532	1.869
**Triglyceride, mmol/L**	−0.026	0.233	0.975 (0.879, 1.081)	0.629	2.154
**Total cholesterol, mmol/L**	0.274	1.759	1.315 (0.877, 1.970)	0.185	2.373
**PCT, ng/mL**	0.060	5.131	1.062 (1.008, 1.118)	0.024	1.284
**CRP, mg/L**	0.005	2.159	1.005 (0.998, 1.011)	0.142	1.425

AKI, acute kidney injury; AP, acute pancreatitis; NRBC, nucleated red blood cells; PT, prothrombin time; APTT, active partial thromboplastin time; RDW-CV, red blood cell volume distribution width – coefficient of variation; RDW-SD, red blood cell volume distribution width-standard deviation; AST, aspartate aminotransferase; CRP, C-reactive protein; PCT, procalcitonin; VIF, variance inflation factor.

Univariate analysis also showed that NRBC count, PT, APTT, D-dimer, PLT, AST, TP, ALB, Hb, RDW-SD, TB, DB, LDL, PCT, and CRP levels were significantly associated with poor prognosis in AP patients. The VIFs were below the conservative threshold of 10 (range = 1.088–3.945), indicating a low risk of multicollinearity that did not pose a threat to the interpretation of the regression coefficients (Table 4). Binary logistic regression analysis indicated that NRBC count (OR = 204.434, p < 0.05), PCT (OR = 1.076, p < 0.05) and CRP level (OR = 1.011, p < 0.05) were independent predictors of poor prognosis in AP patients, and higher levels of the indicators suggested poorer prognosis (Table 4).

**Table 4:** Binary logistic regression analysis for factors associated with poor prognosis in AP patients.

Variables	B	Wald	OR (95% CI)	p – value	VIF
**NRBC, × 10** ^**9**^ **/L**	5.320	7.286	204.434 (4.294, 9733.855)	0.007	2.115
**PT, s**	−0.077	1.635	0.926 (0.824, 1.042)	0.201	1.597
**APTT, s**	−0.014	0.177	0.986 (0.921, 1.054)	0.674	2.345
**D-dimer, μg/mL FEU**	−0.229	2.665	0.796 (0.604, 1.047)	0.103	2.108
**Platelet count, × 10** ^**9**^ **/L**	−0.008	1.349	0.992 (0.979, 1.005)	0.246	1.298
**AST, U/L**	0.004	0.991	1.004 (0.996, 1.012)	0.320	1.394
****Total protein**, g/L**	−0.048	0.430	0.954 (0.827, 1.099)	0.512	1.850
**Albumin, g/L**	−0.065	0.753	0.937 (0.808, 1.086)	0.386	1.806
**Hemoglobin, g/L**	−0.015	0.332	0.985 (0.937, 1.036)	0.564	1.088
**RDW-SD, fl**	0.050	0.244	1.052 (0.861,1.285)	0.621	1.261
**Total bilirubin, μmol/L**	−0.004	0.045	0.996 (0.963, 1.031)	0.833	3.867
**Direct bilirubin, μmol/L**	0.009	0.242	1.009 (0.975, 1.043)	0.622	3.945
**LDL, mmol/L**	−0.364	1.522	0.695 (0.390, 1.239)	0.217	1.707
**PCT, ng/mL**	0.073	5.404	1.076 (1.011, 1.144)	0.020	1.242
**CRP, mg/L**	0.011	7.345	1.011 (1.003, 1.019)	0.007	1.405

AP, acute pancreatitis; NRBC, nucleated red blood cells; PT, prothrombin time; APTT, active partial thromboplastin time; RDW-SD, red blood cell volume distribution width-standard deviation; AST, aspartate aminotransferase; LDL, low-density lipoprotein; CRP, C-reactive protein; PCT, procalcitonin; VIF, variance inflation factor.

### NRBC count predicts AKI and poor prognosis in AP patients

**Fig 3:**
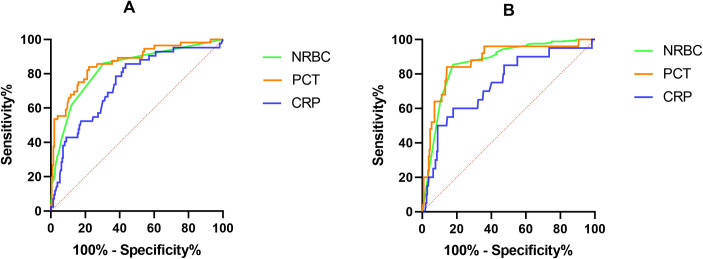
ROC curves for NRBC count, PCT level, and CRP level. ROC, receiver operator characteristics; NRBC, nucleated red blood cell; PCT, procalcitonin; CRP, C-reactive protein; AKI, acute kidney injury; AP, acute pancreatitis. (A) ROC curve for AKI; (B) ROC curve for poor prognosis.

**Fig 4:**
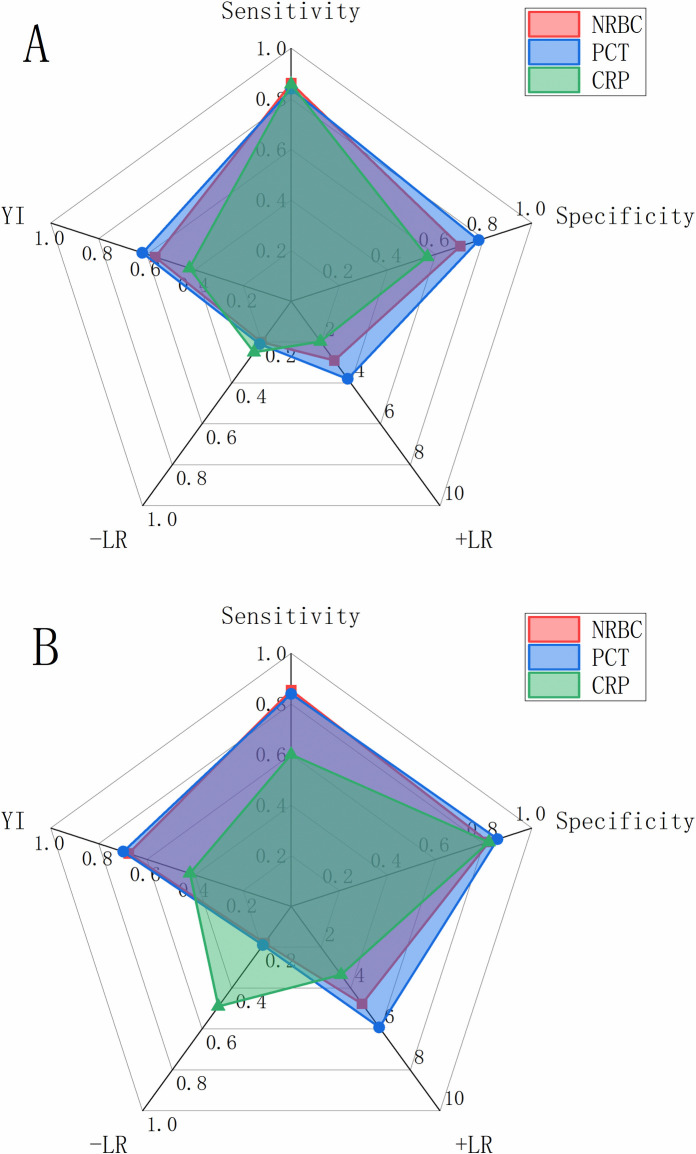
Radar charts of the diagnostic accuracy measures derived from ROC analysis for NRBC count, PCT level, and CRP level. ROC, receiver operator characteristics; NRBC, nucleated red blood cell; PCT, procalcitonin; CRP, C-reactive protein; + LR, positive likelihood ratio; -LR, negative likelihood ratio; YI, maximum Youden’s index. (A) Radar chart of the diagnostic accuracy measures for AKI incidence; (B) Radar chart of the diagnostic accuracy measures for poor prognosis in AP patients.

ROC curves were drawn to explore the performance of NRBC count, PCT and CRP levels in predicting AKI (Fig 3). AUC was computed as 0.830 for NRBC count, 0.864 for PCT level, and 0.745 for CRP level (Table 5). Based on DeLong’s test for AUC comparison, NRBC count outperformed CRP level in predicting AKI (p < 0.001), whereas no significant difference was noted between NRBC count and PCT level. According to the maximum Youden’s index, the cutoff value for NRBC count was greater than 0/L, with a sensitivity of 86.2% and a specificity of 70.3% (Fig 4, Table 5). NRI and IDI were calculated to analyze the enhancement of NRBC count in prediction of AKI (Table 6). As compared to CRP, the NRI and IDI of NRBC count were 0.173 (95%CI: 0.052 to 0.294; p = 0.005) and 0.885 (95% CI: 0.557 to 1.210; p < 0.001), respectively, indicating significant enhancement of NRBC count in prediction of AKI. However, no significant enhancement was found as compared to PCT.

**Table 5:** Comparison of performance of NRBC count, PCT and CRP levels in predicting AKI and poor prognosis in AP patients.

	AKI			Poor prognosis		
	NRBC	PCT	CRP	NRBC	PCT	CRP
**AUC**	0.830 (0.773, 0.887)*	0.864 (0.806, 0.922)*	0.745 (0.664,0.827)* ^#^	0.867 (0.788,0.945)*	0.864 (0.797,0.951)*	0.739 (0.620,0.859)* ^#^
**Cutoff**	0	1.03	88.5	0.015	2.84	214.0
**Sensitivity**	86.2%	83.9%	85.7%	85.4%	84.0%	60.0%
**Specificity**	70.3%	77.9%	56.6%	82.1%	85.8%	82.1%
**+LR**	2.90	3.80	1.97	4.77	5.92	3.35
**-LR**	0.20	0.21	0.25	0.18	0.19	0.49
**YI**	0.565	0.618	0.423	0.675	0.698	0.421

AKI, acute kidney injury; AP, acute pancreatitis; NRBC, nucleated red blood cells; CRP, C-reactive protein; PCT, procalcitonin; AUC, the area under the receiver operating characteristic curves (ROC); + LR, positive likelihood ratio; -LR, negative likelihood ratio; YI, maximum Youden’s index.

* = p < 0.05, as compared to the AUC of the reference line in the ROC analysis. ^#^ = p < 0.05 compared with the AUC of the NRBC according to the DeLong’s test. The optimal cutoff value was determined by the maximum Youden’s index.

**Table 6:** The NRI and IDI of NRBC count compared with PCT and CRP levels in predicting AKI and poor prognosis in AP patients.

		NRI (95%CI)	p – value	IDI (95%CI)	p – value
**AKI**	**NRBC + PCT**	−0.071 (−0.278, 0.136)	0.504	−0.110 (−0.260, 0.040)	0.151
	**NRBC + CRP**	0.173 (0.052, 0.294)	0.005	0.885 (0.557, 1.210)	<0.001
**Poor prognosis**	**NRBC + PCT**	0.061 (−0.127, 0.249)	0.523	−0.018 (−0.196, 0.160)	0.842
	**NRBC + CRP**	0.522 (0.050, 0.994)	0.030	0.951 (0.489, 1.410)	<0.001

NRI, net reclassification index; IDI, integrated discrimination improvement; NRBC, nucleated red blood cells; PCT, procalcitonin; CRP, C-reactive protein; AKI, acute kidney injury; AP, acute pancreatitis; CI, confidence interval.

**Fig 5:**
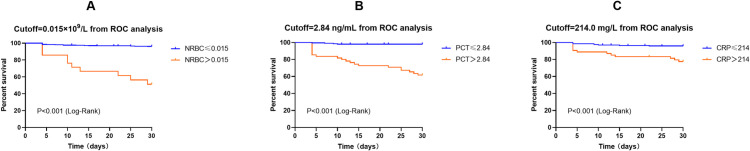
Kaplan–Meier survival curves. NRBC, nucleated red blood cell; PCT, procalcitonin; CRP, C-reactive protein.

Similarly, the performance of NRBC count, PCT and CRP levels in predicting poor prognosis in AP patients was analyzed using ROC curves (Fig 3). AUC values for NRBC count, PCT and CRP levels were 0.867, 0.864, and 0.739, respectively (Table 5). Through AUC comparison, no significant difference was noted between NRBC count and PCT level in predicting poor prognosis in AP patients, while NRBC count was superior to CRP level (p < 0.001). According to the maximum Youden’s index, the cutoff value for NRBC count was 0.015 × 10^9^/L, with 85.4% sensitivity and 82.1% specificity (Table 5). ROC-derived diagnostic accuracy measures for NRBC count, PCT, and CRP levels were summarized in Table 5 and Fig 4. As compared to CRP level, NRBC count demonstrated significant enhancement in predicting poor prognosis in AP patients (NRI = 0.522, 95%CI: 0.050–0.994, p = 0.030; IDI = 0.951, 95%CI: 0.489–1.410, p < 0.001); however, no significant enhancement was observed when compared to PCT (Table 6). According to the optimal cutoff value of NRBC count (0.015), PCT level (2.84) and CRP level (214.0), patients were further stratified into two subgroups, respectively. Kaplan–Meier survival analysis with log-rank tests demonstrated that patients with NRBC count > 0.015 (χ² = 85.09, p < 0.001), PCT > 2.84 (χ² = 71.17, p < 0.001) or CRP > 214.0 (χ² = 20.07, p < 0.001) exhibited significantly lower survival rates during the 30-day follow-up period (Fig 5).

## Discussion

AKI is a frequent complication in patients with AP, severely affecting the clinical outcomes of the patients. In our study, the incidence of AKI in AP patients is about 32.5%. As reported, the incidence of severe acute pancreatitis (SAP) combined with AKI can be up to 69.3% [15]. A current diagnosis of AKI essentially relies on Crea elevation. However, a significant increase in the serum Crea level fails to achieve an early identification of AKI [16].

This study, for the first time, demonstrates that NRBC count can be used as a novel biomarker for predicting AKI incidence and poor prognosis in patients with AP. We found that AP patients with NRBCs present in the peripheral blood were prone to experience a severer disease, higher incidence of AKI, extended hospital stays, increased reliance on hemopurification, and a greater likelihood of unfavorable prognosis. Binary logistic regression also showed that NRBC count was an independent predictor for both AKI and poor prognosis in AP patients. ROC curve analysis revealed that NRBC count possessed the potential in early diagnosis of AKI and prediction of poor prognosis in AP patients. Moreover, Kaplan-Meier survival analysis indicated that the elevation of NRBC count (cutoff: > 0.015 × 10⁹/L) was associated with poor survival outcomes, reinforcing their prognostic utility in identifying high-risk AP patients.

Among the existing biomarkers of AP-AKI, NRBC count demonstrates distinct advantages. Unlike Crea with delayed response, NRBC count may reflect systemic hypoxia or inflammation [1,2,17–21], and NRBCs are released into the circulation during the early pathophysiological stages of AKI. In this study, the performance of NRBC count in AKI prediction (AUC = 0.830) was comparable to PCT (AUC = 0.864) and significantly superior to CRP (AUC = 0.745; NRI = 0.173, IDI = 0.885). Thus, NRBC count has the potential to predict early AKI in AP patients, and it also demonstrates comparable predictive performance in prognosis. Moreover, NRBC counting relies on routine blood analysis and requires no additional costs or specialized equipment, making it particularly suitable for resource-limited primary care settings. In contrast, PCT testing depends on immunoassay technology, which is costly and not widely accessible. Other markers, such as neutrophil gelatinase-associated lipocalin (NGAL), kidney injury molecule 1 (KIM-1), and interleukin-18 (IL-18), have shown promise for early AKI diagnosis [22–26]. However, their application in AP-related AKI remains understudied, and their complex measurement protocols hinder widespread clinical adoption.

However, NRBC count as a biomarker for AP-AKI has some limitations. First, an elevation of NRBCs is not specific to AKI and may be influenced by other conditions such as severe anemia and hematologic malignancies, potentially leading to false-positive interpretations. Second, current evidence supporting the role of NRBC count in AP-AKI primarily derives from our single-center study with limited sample sizes, necessitating validation in larger, multicenter cohorts to confirm generalizability. Third, the precise pathophysiological mechanisms underlying the role of NRBCs in AP-AKI remain unclear.

The pathophysiology of AP-AKI remains elusive, potentially related to hypovolemia and subsequent complex interplay between inflammatory, vascular, and humoral factors. Under normal conditions, NRBCs are usually found in the peripheral blood of newborns and pregnant women. While in other populations with NRBCs present in the peripheral blood, severe stress or pathologic erythropoiesis is usually considered [17–21,27–30]. Systemic inflammation, hypoxia, and massive hemorrhage can lead to increased erythropoietic stress, resulting in increased NRBCs [17–21]. This study excluded pregnant women and patients with possible hematologic disorders. We assume that hypoxia and inflammation may play a part in the generation and release of NRBCs. Hypovolemia-induced tissue hypoperfusion in early AP-AKI activates hypoxia-inducible factor (HIF), which promotes erythropoietin (EPO) production and accelerates erythroid progenitor maturation in the bone marrow [31]. This compensatory mechanism aims to enhance oxygen delivery but may lead to premature NRBC release into the circulation [32,33]. This study found an elevation of plasma lactate levels (a marker of tissue hypoxia) in NRBC (+) patients (3.11 (2.46, 3.64) vs. 1.77 (1.34, 2.25) mmol/L, p = 0.002), which supports this hypothesis. Moreover, systemic inflammation in AP disrupts normal erythropoiesis. Pro-inflammatory cytokines, such as interleukin-1β (IL-1β) and tumor necrosis factor-α (TNF-α), suppress erythroid maturation in the bone marrow and promote the expansion and differentiation of stress erythroid progenitors in the spleen [34], potentially resulting in increased release of immature NRBCs [19,35,36]. Consistently, this study found an elevation of WBC count, neutrophil count, PCT, and CRP levels in NRBC (+) patients, suggesting hematopoietic stress response mediated by inflammatory mediators. Additionally, the interplay between hypoxia and inflammation can create a pathologic feedback loop. Hypoxia exacerbates pro-inflammatory cytokine production [37–40], while inflammatory mediators impair oxygen utilization [41,42]. This “hypoxia-inflammation axis” may explain why NRBC (+) AP patients exhibit severer organ dysfunction than NRBC (-) AP patients. However, further research is required to verify the above-mentioned hypothesized mechanism.

This study has several drawbacks. First, this study was a single-center retrospective cohort study, which may have introduced bias. Second, this study only focused on the NRBC count on admission but neglected its role in long-term disease progression. These limitations highlight the need for further investigation in future studies.

## Conclusions

In summary, NRBC count serves as a pivotal predictive biomarker for both AKI and prognosis in AP patients.

## Supporting information

S1 File The Original Data. (XLS)
